# Unlocking the Potential of Stroke Blood Biomarkers: Early Diagnosis, Ischemic vs. Haemorrhagic Differentiation and Haemorrhagic Transformation Risk: A Comprehensive Review

**DOI:** 10.3390/ijms241411545

**Published:** 2023-07-17

**Authors:** Lazzaro di Biase, Adriano Bonura, Pasquale Maria Pecoraro, Simona Paola Carbone, Vincenzo Di Lazzaro

**Affiliations:** 1 Fondazione Policlinico Universitario Campus Bio-Medico, Via Alvaro del Portillo, 00128 Roma, Italy; adriano.bonura@unicampus.it (A.B.); p.pecoraro@unicampus.it (P.M.P.); s.carbone@unicampus.it (S.P.C.); v.dilazzaro@unicampus.it (V.D.L.); 2 Brain Innovations Lab, Università Campus Bio-Medico di Roma, Via Álvaro del Portillo 21, 00128 Rome, Italy; 3 Unit of Neurology, Neurophysiology, Neurobiology and Psychiatry, Department of Medicine and Surgery, Università Campus Bio-Medico di Roma, Via Alvaro del Portillo, 00128 Roma, Italy

**Keywords:** blood biomarkers, stroke early diagnosis, stroke differential diagnosis, stroke prognosis, predictive stroke biomarkers, ischemic stroke, haemorrhagic stroke

## Abstract

Stroke, a complex and heterogeneous disease, is a leading cause of morbidity and mortality worldwide. The timely therapeutic intervention significantly impacts patient outcomes, but early stroke diagnosis is challenging due to the lack of specific diagnostic biomarkers. This review critically examines the literature for potential biomarkers that may aid in early diagnosis, differentiation between ischemic and hemorrhagic stroke, and prediction of hemorrhagic transformation in ischemic stroke. After a thorough analysis, four promising biomarkers were identified: Antithrombin III (ATIII), fibrinogen, and ischemia-modified albumin (IMA) for diagnostic purposes; glial fibrillary acidic protein (GFAP), micro RNA 124-3p, and a panel of 11 metabolites for distinguishing between ischemic and hemorrhagic stroke; and matrix metalloproteinase-9 (MMP-9), s100b, and interleukin 33 for predicting hemorrhagic transformation. We propose a biomarker panel integrating these markers, each reflecting different pathophysiological stages of stroke, that could significantly improve stroke patients’ early detection and treatment. Despite promising results, further research and validation are needed to demonstrate the clinical utility of this proposed panel for routine stroke treatment.

## 1. Introduction

“Time is brain” reflects intervention timing relevance in stroke. Pathophysiological terms reflect this in the loss of 1.9 million neurons and 14 billion synapses approximately every minute after stroke onset [1,2]. In clinical terms, it is embodied in an increased risk of long-term disability, in-hospital mortality, and intraparenchymal haemorrhage for every 15-min delay in therapy administration [1,3,4]. Indeed, acute phase therapy is represented by revascularization therapies, such as thrombolysis and thrombectomy [5,6,7,8]. The efficacy and safety of these therapies are strictly time-dependent. Pooled analyses from the ECASS [9], ATLANTIS [10], NINDS [11], and EPITHET [12] trials show that a delay in the start of treatment (OTT) is associated with a reduction in 90-day disability and mortality [13]. Anyway, in clinical practice, timely therapeutic intervention is not always possible. Several factors may cause a delay in the door to treatment time, such as failure to recognize stroke symptoms and signs, delay in ambulance transport, inappropriate triage, and difficulty in performing CT imaging [14].

Indeed, imaging methods such as CT brain are fundamental in the first diagnostic phase of stroke as they exclude intracranial haemorrhage (ICH), which represents the main contraindication to the revascularization therapies administration [5,7,8]. In the early stages of stroke, however, CT does not allow for assessing the actual presence of an ischemic lesion, as the first signs usually begin to appear after 12 h from onset [15]. Although MRi with diffusion-weighted imaging (DWI) sequences can highlight an ischemic lesion already in the first hours [16], the execution in acute cases is not always possible for economic and logistic reasons (high cost of equipment, availability in all hospitals and timing of the examination) [17]. Often no examination objectifies the presence of an acute stroke, and the differential with stroke mimics.

In recent years, the search for biological markers that can diagnose stroke early and differentiate the various types (ischemic and haemorrhagic) following the steps made in myocardial infarction with troponin I [18] has become a hot topic. Although several biomarkers have been proposed [19,20], finding molecules that guarantee high diagnostic accuracy and early diagnosis of stroke remains a challenge. Stroke is a heterogeneous pathology characterized by different pathophysiological processes that vary from case to case and that may influence the diagnostic performance of a biological marker [21,22]. This review aims to investigate the presence of possible stroke biomarkers in the literature. In particular, the research is focused on three types of biomarkers:

- Diagnostic biomarkers: able to differentiate stroke (ischemic or haemorrhagic) from stroke mimics
- Differentiating biomarkers: able to distinguish intraparenchymal haemorrhage from cerebral ischemia
- Predictive biomarkers: able to predict the possible haemorrhagic transformation of an ischemic stroke.

Finally, a possible biomarkers panel has been discussed to improve stroke diagnosis, give information about the ischemic or haemorrhagic nature of the symptomatology, and give predictive information about a potential risk of haemorrhagic transformation.

## 2. Methods

A MEDLINE (PubMed) literature review was performed to identify all published studies about Stroke (ischemic or haemorrhagic) Biomarkers. The string described in Table 1 was used in the PubMed search engine for article selection based in part on the study by Baez et al. [23].

We included plasma and serum biomarkers for stroke diagnosis, differentiation between haemorrhagic and ischemic stroke, and predictive of haemorrhagic transformation. We selected only studies that presented diagnostic performance indices such as sensitivity, specificity, area under the curve (AUC), positive predictive value (PPV), or negative predictive value (NPV). Articles concerning biomarkers with prognostic function were excluded because they were not in line with the purpose of this review. The various biomarkers were described individually and by collecting the following data: pathophysiology of the marker, number of patients tested, the purpose of the marker (diagnostic, differential or predictive), and diagnostic performance index. Starting from sensitivity and specificity data or their mean value, for each predictor has been calculated the likelihood ratio negative (LR−), likelihood ratio positive (LR+) and the diagnostic odds ratio (DOR).

## 3. Results

**Figure 1:**
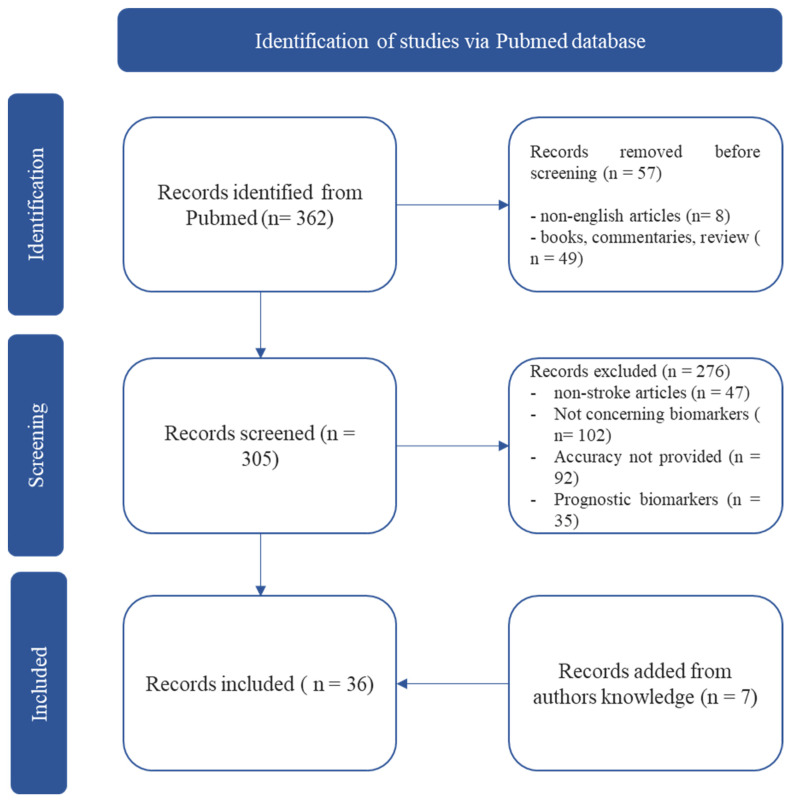
Search strategy flowchart.

We found 362 total articles. Reviews, letters, editorials, commentaries and books or non-English papers were excluded before screening (*n* = 57). After carefully analysing the title and abstract and evaluating inclusion and exclusion criteria, *n* = 29 articles were included in the present review. We excluded *n* = 276 studies based on non-stroke related articles (*n* = 47), articles not concerning biomarkers (*n* = 102), articles without diagnostic accuracy data (*n* = 92) and articles on prognostic stroke biomarkers (*n* = 35). Finally, we add *n* = 7 articles from the authors’ knowledge Figure 1.

### 3.1. GFAP

Glial fibrillary acidic protein (GFAP) is a proteolytic enzyme expressed in astrocytes [24]. It is involved in various processes, from cell-cell communication and astrocyte-neuron interaction [25] to maintenance of the blood-brain barrier [26]. It is also associated with post-injury reparative processes in the central nervous system [27]. The rupture of astrocytic cells that occurs during intraparenchymal haemorrhage would result in a rapid release of GFAP into the blood, in contrast to a slower GFAP blood increase in acute cerebral ischemia [28]. These characteristics make it a possible biomarker for ischemic and haemorrhagic stroke differential. Foerch et al. conducted two studies on GFAP [28,29]. First, the diagnostic accuracy of GFAP in recognizing intracranial haemorrhage was evaluated in 135 stroke patients (42 ICH, 93 IS) within six h of symptom onset. The results showed for values > 2.9 ng/L a sensitivity of 79% and specificity of 98%, a positive predictive value (PPV) of 94% and a negative predictive value (NPV) of 91% for the identification of ICH [28]. The other study was conducted on 205 patients, 39 with ICH, 163 with ischemic stroke, and three stroke mimics. Results showed that GFAP concentrations were higher in ICH than in ischemic stroke (1.91 μg/L (0.41–17.66) vs. 0.08 μg/L (0.02–0.14). Again, the cut-off was 2.9 ng/mL showing a sensitivity of 84.2% and a specificity of 96.3% in differentiating ICH from IS and stroke mimics. Blood sampling was performed with a mean time from symptom onset of 125 min [29]. Another study showed that the sensitivity of GFAP in detecting ICH depended largely on the size of the haemorrhage. Blood samples from 74 stroke patients (25 ICH, 49 IS) were analysed with a GFAP cut-off = 0.29 ng/mL. Nine ICH patients had GFAP levels above the cut-off. The sensitivity was 36%, and the specificity was 100%. Sensitivity was 61.5% when assessed against an ICH volume > 15 mL, showing that GFAP increases as a function of hematoma size [30]. GFAP has been associated with retinol-binding protein 4 in differentiating ICH from IS. IS patients with RBP4 > 48.75 lg/mL and GFAP < 0.07 ng/mL were differentiated from ICH with a sensitivity = 68.4%, specificity = 84%, positive predictive value (PPV) = 86.7% and negative predictive value (NPV) = 65.6%. For RBP4 values > 61 mcg/mL and GFAP < 0.07 ng/mL, the specificity in discerning between the two subtypes of Stroke is 100% [31]. A cut-off of 0.7 ng/mL showed a sensitivity of 86% and specificity of 76.9%, confirming the relationship between GFAP levels and disability [32].

A study of 270 subjects with acute neurological symptoms (AIS: 121, ICH: 34, stroke mimics: 31, subarachnoid haemorrhage: 5, controls: 79) for cut-off values of 0.43 ng/mL GFAP (taken within 6 h of symptom onset) showed a sensitivity of 91% and specificity of 97% (AUC 0.97) in differentiating ICH from AIS [33].

Finally, two meta-analyses showed GFAP’s ability to differentiate ICH from IS and mimic with a sensitivity of 78% and 75% and a specificity of 95% (AUC: 0.93 and 0.9) [34,35].

### 3.2. NSE

Neuron-specific enolase (NSE) is expressed in mature neurons and neuron-derived cells. It is a key metalloenzyme in glycolysis. Small cell carcinomas also produce it with a neuroendocrine origin and are a marker for differentiating such tumors [36]. Neuronal death results in the release of NSE into the blood. Elevated blood NSE values have been associated with higher volume strokes [37]. In addition, monitoring NSE values in stroke patients may be useful in predicting haemorrhagic transformation of acute cerebral ischemia [38]. Rising NSE values and the presence of a second peak result in an increased risk of haemorrhagic transformation (odds ratio = 6.844), according to a study of 83 stroke patients [38]. NSE has shown low diagnostic accuracy in early stroke detection. In a study performed on 79 patients (44 ischemic, 17 haemorrhagic, and 11 mimic strokes) for cut-off values ≤ to 14 mcg/L, the sensitivity was 53% and specificity 64% in differentiating strokes versus mimics [39].

### 3.3. S100b

S100 calcium-binding protein B is a glial protein expressed in mature perivasal astrocytes [40]. Among other functions, this protein allows neurite extension, astrocyte proliferation, and inhibition of microtubule assembly [41]. The presence of neuronal and blood-brain barrier damage results in astrocyte activation and release into the bloodstream of S100b [42].

S100B levels are increased in haemorrhagic stroke compared to ischemic stroke in several studies, thus allowing a distinction between the two forms of brain damage. A cut-off of 96 pg/mL of S100b associated with sRAGE < 0.97 ng/mL was evaluated in one study. The two molecules combined showed a specificity of 80.2% and a sensitivity of 22.7%. However, when combined with clinical parameters such as male sex, age, prior haemorrhagic stroke, coronary artery disease, and NIHSS on admission, they show an AUC of 0.77. (CI 95% 0.70–0.84) [43]

S100B showed a sensitivity of 55% and specificity of 64% in differentiating between stroke and stroke mimics for cut-off values <= to 130 ng/L [39]. In another study, four biomarkers were evaluated (BNP, MMP-9, S100B and D-dimer), showing a sensitivity of 90% in predicting all types of strokes (sensitivity 90%, specificity 45%), intracranial haemorrhage (sensitivity 88%, specificity 38%), and ischemic stroke (sensitivity 91%, specificity 45%) within 3 h of symptom onset. S100b, among the markers evaluated, proved to be the one that contributed the least to the diagnostic accuracy of the model [44]. S100b was also evaluated in a panel of molecules (BNP, D dimer, MMP9, and S100B) to diagnose acute stroke showing an AUC of 0.712 [45]. Kazmierski et al. conducted a study on 458 stroke patients evaluating the efficacy of different biomarkers in predicting haemorrhagic transformation in IS patients. The S100b showed a sensitivity of 92.9%, a specificity of 48.1%, a PPV of 12.2%, and an NPV of 98.9% with an AUC of 0.746 [46].

One study evaluated S100A12, a mediator of inflammatory response with RAGE receptor [47] that appears to have increased levels in patients with atherosclerosis and recent symptoms of stroke [48]. In a study of 167 patients (57 ischemic, 32 ICH, 41 TIA, and 37 mimics) at 4.5 h after symptom onset, S100A12, together with eotaxin, egfr, MMP 4, and prolactin showed a sensitivity of 90% and specificity of 84%, PPV 78%, and NPV 93% in differentiating strokes (ischemic and haemorrhagic) from mimics [48].

### 3.4. MMP-9

Metalloproteinase 9 (MMP-9) is a zinc-metalloproteinase involved in extracellular matrix degradation [49]. During a stroke, disruption of the endothelial layer results in the release of molecules such as MMP-9, E-selectin, P-selectin, VonWillebrand factor, and exposure of the extracellular matrix [50]. The increase in the bloodstream of MMP-9 seems to be associated with the haemorrhagic transformation of an ischemic stroke [46,51,52]. In a study of 168 stroke patients, MMP-9 at a concentration > 181.7 ng/mL showed a sensitivity of 82.9%, a specificity of 81.3%, a PPV of 48%, and an NPV of 95.8% in predicting haemorrhagic transformation [51]. Blood samples were collected 1 h after admission in the hospital within 24 h after symptoms onset. In Kazmierski’s study, the predictive abilities of MMP9 showed a sensitivity of 68.7%, a specificity of 45.3%, a PPV of 8.9%, and an NPV of 94.9% [46]. As mentioned above, MMP-9 associated with D-dimer, BNP and S100b showed good sensitivity in the diagnosis of stroke and the differentiation between the various forms of stroke [44,45]. Finally, a meta-analysis conducted on 12 studies and 1492 patients showed a sensitivity of 85% (95% CI: 75%, 91%), a pooled specificity of 79% (95% CI: 67%, 87%), with significant heterogeneity (*p* < 0.05, I^2^ = 53.51% and *p* < 0.01, I^2^ = 97.94%) and AUC of the HSROC of 0.89 in predicting haemorrhagic transformation of ischemic stroke [52].

### 3.5. NT-ProBNP

Brain natriuretic peptide (BNP) is secreted by cardiac ventricles in response to cardiomyocyte elongation. It is secreted as a prohormone bound to an N-terminal fragment called NT-proBNP that is biologically inactive. Once activated, it determines various effects: at the renal level, it increases the filtration rate at a vascular level, where it determines vasodilation and at the cardiac level, where it has an anti-fibrotic effect [53]. High levels of BNP in the blood have been associated with heart failure and atrial fibrillation [54]. For this reason, BNP or NT-proBNP may be a biomarker of cardioembolic stroke. The results of a study conducted on 92 patients (66 with ischemic stroke) in which NT-proBNP was assessed within 72 h after stroke showed a cut-off of 265.5 pg/mL, sensitivity and specificity in diagnosing cardioembolic stroke of 71.4% and 73.7% respectively with AUC values = 0.77 [55]. In the diagnosis of cardioembolic stroke associated with atrial fibrillation, the AUC was 0.92, with a sensitivity of 94.4% and a specificity of 72.9% [55]. The stroke chip study [56] analysed the diagnostic accuracy of 21 biomarkers of stroke diagnosis in the differentials between ischemic and haemorrhagic strokes. The biomarkers that showed better diagnostic accuracy were NT pro-BNP (sensitivity 76.9%, specificity 43.5%) and IL 6 (sensitivity 76.8% and specificity 40.7%) in diagnosing stroke vs. mimics. Regarding the ability to discern between ischemic and haemorrhagic stroke, NT proBNP showed a sensitivity of 44.8% and specificity of 74.9%. In contrast, biomarkers such as IL 6, D-dimer and endostatin showed high specificity but sensitivity < 30% [56].

### 3.6. IMA and IMA Index

Hypoxia, acidosis, and oxygen radical damage can alter the N-terminal portion of albumin, reducing its metal-binding capacity and forming ischemia-modified albumin (IMA) [57,58]. IMA is an early biomarker of myocardial ischemia, becoming positive 6–10 min after ischemia and remaining elevated for 6 h [59,60]. The diagnostic accuracy of IMA in stroke has been evaluated in several studies. Abboud et al. studied IMA levels in 118 patients (84 strokes, 18 haemorrhage, 16 TIA or epilepsy) within 3 h of symptom onset. The optimal diagnostic cut-off was 80 U/mL, with a sensitivity of 57.8% and specificity of 81.3%. The corresponding PPV, NPV, LR+, and LR− were 91.7%, 21.7%, 1.73, and 0.56, respectively [61]. In another study, IMA showed a sensitivity of 86.8% and a specificity of 60.5% in detecting subarachnoid haemorrhage, ischemic stroke, and haemorrhagic stroke [62]. Ahn et al. evaluated the efficacy of the IMA index = serum albumin concentration (g/dL) × 23 + IMA (U/mL) − 100 in stroke diagnosis. Fifty-two patients with neurologic symptoms (28 ischemic strokes and 24 non-strokes) were evaluated. Samples were taken within 3 h of symptom onset, showing a sensitivity of 95.8% and specificity of 96.4% with an AUC of 0.990 for a cut-off of 98 U/mL [63].

### 3.7. FABP

Fatty acid binding proteins (FABP) are a family of cytoplasmic proteins involved in the metabolism of fatty acids, ensuring their transport to the mitochondrion for oxidation [64]. Hearth-fatty acid binding protein (h-FABP) is present within myocardiocytes and represents an early biomarker of myocardial infarction [64]. H-FABP is also contained in neurons, so it has been proposed as a potential biomarker in early stroke diagnosis. Zimmermann et al. [65] analysed h-FABP, troponin I, and CK-MB levels in 64 patients (22 controls, 22 stroke patients, and 20 patients with acute myocardial infarction). Levels of h-FABP were increased in patients with stroke and patients with myocardial infarction, whereas troponin I and CK-MB values were increased only in patients with myocardial infarction. The sensitivity of h-FABP in the diagnosis of stroke was 68.2%, with a specificity of 100% for optical density cut-off values (OD value) > 0.531 [65]. In another study, HFABP was compared with S100b in the diagnosis of stroke and in the prediction of long-term clinical outcomes showing a sensitivity of 59.5%, specificity of 79.5% and AUC 0.71. Blood levels also correlate with initial NIHSS and clinical outcomes [66]. The brain-fatty binding protein (b-FABP) contained in astrocytes has also been proposed as a potential biomarker in the diagnosis of stroke, but no articles have been found specifying its diagnostic value in terms of sensitivity, specificity and AUC [67].

### 3.8. NR2 and Anti-NR2A/B Antibodies

The NR2 peptide is a degradation product of the N-methyl-D-aspartate (NMDA) receptor. During the acute phase of the ischemic cascade, a massive glutamate release causes excitotoxicity on NMDA receptors. This process seems to lead to cleavage of the NR2 subunit by serine proteases and release into the bloodstream [68]. A study of the potential efficacy of NR2 as a biomarker in diagnosing stroke and the differential between stroke and stroke mimics was conducted. One hundred ninety-two patients with suspected stroke or TIA were enrolled, performing blood draws within 72 h of symptom onset and a brain MRI 24 h after hospital arrival. Results showed a sensitivity of 92%, specificity of 96%, and a PPV of 93% for a cut-off value of 1.0 μg/L of NR2 [69]. The efficacy as a biomarker of antibodies to NR2A/B (NMDA subunits) was evaluated in 205 patients with stroke (ischemic and haemorrhagic) and TIA and in 255 controls (with risk and non-risk factors). Sampling was performed on average between 3–5 h in ICH and 9–12 h in IS. For cut-off values of 2.0 ng/mL, sensitivity was 98%, specificity 97% in stroke and 95% in TIAs with an AUC 0.99 in the differential between stroke/TIA and controls. Lower values with an earlier peak of anti-NR2A/B were found in patients with ICH, compared with patients with IS [70].

### 3.9. ATIII and Fibrinogen

Antithrombin III (ATIII) is synthesized by the endothelium of blood vessels and the liver in a 1:1 ratio with thrombin. A reduction in blood values represents an index of activation of the coagulation cascade [71]. In a study of 198 patients (152 IS and 46 stroke mimics), ATIII showed a sensitivity of 97.32% and a specificity of 93.62% for cutoff values < 210% in differentiating stroke from stroke mimics. Samples were taken within 4.5 h of symptom onset. Fibrinogen was assessed in the same study, which showed a sensitivity of 96.05% and specificity of 82.61% for values > 4 g/L [72].

### 3.10. Other Biomarker

Following a description of other biomarkers still poorly studied in stroke diagnosis.

**Adrenomedullin** (AM) is a vasodilator peptide hormone used as a pheochromocytoma biomarker [73]. Studies showed that AM expression is upregulated under hypoxia via activation of the hypoxia-inducible factor-1 (HIF-1) pathway [74], and blood levels increase after ischemic brain insult [75]. Moreover, blood AM levels were used as a diagnostic marker of intracranial haemorrhage. Blood samples were taken at admission, after 24 h, and after seven days. The study enrolled 114 patients (50 controls and 64 with intracranial haemorrhage) and showed increased AD levels in patients with haemorrhagic stroke compared with controls from the first blood draw. For an AD value > 69 pg/mL, the sensitivity was 80%, specificity 100% and AUC 0.89. Reduced AD levels after admission are also associated with better functional recovery [76].

**Homocysteine** is an amino acid derived from methionine. Elevated blood levels are considered a cardiovascular risk factor. Wang et al. investigated the association between total plasma homocysteine levels and the risk of early haemorrhagic transformation in patients with ischemic stroke. The results showed a sensitivity of 63.3% and a specificity of 41.3% for homocysteine levels > 16.56% [77].

**MicroRNA (miR)** is a non-coding RNA that regulates gene expression, cell development, apoptosis and metabolism [78]. Some miRNAs are tissue-specific: such as the brain-specific miR-124-3p, which is shown to be a biomarker of brain damage in mice [79]. The miR 16 is involved in apoptosis and appears to play a role in brain ischemia [80]. Plasma concentrations of **miR 124-3p** and **miR-16** to discriminate haemorrhagic and ischemic stroke within 24 h were evaluated in one study. For miR 124-3p levels > 3 × 10^5^ copies/mL, sensitivity and specificity were 68.4% and 71.2%, respectively. For plasma levels of miR, 16 cutoffs ≤ 2 × 10^9^ copies/mL, sensitivity and specificity were 96.7% and 35.1%, respectively [81].

A study of changes in small metabolites in blood and urine with proton nuclear magnetic resonance of stroke patients showed increased levels of lactate, pyruvate, glycolate, and formate and reduced levels of glutamin and methanol in plasma; conversely, reduced levels of citrate, hippurate, and glycine were found in the urine of stroke patients [82]. Based on these findings, a study was performed to evaluate whether **metabolomics** in stroke may allow the identification of potential diagnostic biomarkers. 11 biomarkers included hydroxylbutyrylcarnitine, glutarylcarnitine (C5DC), myristoylcarnitine, 3-hydroxypalmitoylcarnitine, tyrosine/citrulline (Cit), valine/phenylalanine, C5DC/3-hydroxyisovalerylcarnitine, C5DC/palmitoylcarnitine, hydroxystearoylcarnitine, the ratio of the sum of C0, C2, C3, C16, and C18: 1 to Cit, and propionylcarnitine/methionine were evaluated for the differential between ischemic and haemorrhagic stroke. The biomarker panel showed a sensitivity of 84% and a specificity of 76.9% [83].

**Interleukin 33 (IL-33)** is a protein member of the interleukin family produced by T helper2. It is expressed in fibroblasts, mast cells, dendritic cells, macrophages, and endothelial cells [84]. IL-33 is an independent predictor of haemorrhagic transformation in patients with acute cerebral ischemia. The study on 151 patients with IS showed IL 33 values < 67.66 ng/L, a sensitivity of 81.3% and a specificity of 63% with an AUC of 0.739 [85].

Blood-brain barrier tight junction components were evaluated as predictors of haemorrhagic transformation in patients with IS. Molecules such as **Occludin**, **Claudin 5** and **ZO1** showed increased values in patients with haemorrhagic transformation. Occludin showed a sensitivity of 58.6%, specificity of 67.5%, PPV of 12.3% and NPV of 95.5% with an AUC of 0.622. Claudin 5 sensitivity of 64.3%, specificity of 65.8%, PPV 16.8% and NPV 94.5% with AUC 0.599. The ZO1 has a sensitivity of 56.7%, specificity of 56.0% PPV of 9.1%, NPV of 94.3% and AUC of 0.519 [46].

**Tumor necrosis factor-alpha (TNF alpha**) is a cytokine involved in systemic inflammation [86]. It has been used as a biomarker in predicting DWI-positive lesions after carotid stenting. The study showed that for cut-off values of 9.45 pg/mL, the sensitivity of TNF alpha was 45.3%, and the specificity was 82.8% with an AUC of 0.651 [87].

**PARK7,** or **DJ-1,** is a protein that regulates the oxidative stress response and is upregulated in prostate tumors. A mutation in the gene expressing PARK7 is also associated with an autosomal recessive form of early-onset Parkinson’s disease [88,89]. Nucleoside diphosphate kinase (NDKA) is an enzyme responsible for the catalysis of phosphate group exchange between different nucluoside diphosphate groups. Mutations in the gene have been found in patients with Alzheimer’s disease and Down syndrome [90]. It appears to be increased in response to either an ischemic or haemorrhagic brain injury. A study has shown that PARK7 and NDKA are increased at 3 h after stroke with a sensitivity of 54–91% for PARK7 and 70–90% for NDKA and specificity of 80–97% for PARK7 and 90–97% for NDKA [91]. Another study compared PARK7 and NDKA levels in various populations (Swiss, Spanish, and US), showing for PARK7, the sensitivity and specificity were 91 and 80%, respectively. For NDKA, the sensitivity and specificity were 90% [92]. Both biomarkers did not demonstrate differential ability between ischemia and haemorrhage, nor association with severity of brain injury [91].

**Glycogen phosphorylase BB (GP-BB**) is an enzyme that catalyzes the degradation reaction of glycogen to glucose-1-phosphate [93]. This enzyme is activated under hypoxic conditions with abrupt reduction of glycogenolysis [93,94]. It is found at the myocardial level and the cerebral level. It represents an early biomarker of myocardial infarction with rapidly increasing plasma within the first hour of chest pain in over 90% of the patients [95]. In the brain, GP-BB is primarily localized in perivascular astrocytes, so its increase due to damage to the blood-brain barrier determines an early increase in the blood [96]. The possibility of using it as a diagnostic biomarker of stroke was evaluated in a study of 172 strokes and 133 non-stroke patients. Blood samples were taken < 4.5 h after symptom onset. For a 7.0 ng/mL cut-off, sensitivity and specificity were 93%, with a curved ROC of 0.96. To exclude an increase in blood enzyme secondary to myocardial infarction, troponin I was also assayed [97].

### 3.11. Stroke Diagnosis Biomarkers

Diagnostic biomarkers differentiate stroke (haemorrhagic or ischemic) from stroke mimics. An optimal stroke biomarker for screening purposes should be characterized by high sensitivity to find the highest number of real stroke patients in the first hours (<4.5 h).

As previously mentioned, the IMA is an early biomarker of myocardial infarction [59,60]. Ischemia seems to determine the activation of reactive oxygen species that modify the N-terminal portion of albumin since the first minutes [57,58,59,60]. IMA values also increase early in the stroke, where studies have evaluated positivity over the cut-off value within 3 h after symptoms onset [61,62]. However, the greatest diagnostic accuracy is achieved by the IMA index, where albumin is corrected for unmodified albumin. While IMA showed a sensitivity of 57.8% with an LR+ of 3.09, the IMA index showed a sensitivity of 95.8% and an LR+ of 26–61 with a DOR of 610.79 (see Table 2). However, these data come from a single study on a few patients [63].

ATIII represents an ideal biomarker with high diagnostic accuracy in stroke diagnosis with high sensitivity (97.32%), high LR+ (15.25) and DOR (532.86). Furthermore, the conducted study demonstrates an early reduction of ATIII blood values during a stroke (<4.5 h) [72]. This feature depends on the immediate activation of the coagulation cascade due to the rupture of the hematoencephalic membrane, which leads to the consumption of ATIII [71]. In the same study, fibrinogen showed high diagnostic performance (sensitivity: 96.05%, LR+ 5.52, DOR: 115.51).

NDKA also showed good diagnostic performance in stroke detection in the first 3 h from symptoms onset (sensitivity:70–90%, LR+: 12.31 DOR: 57.54) [91,92].

**Figure 2:**
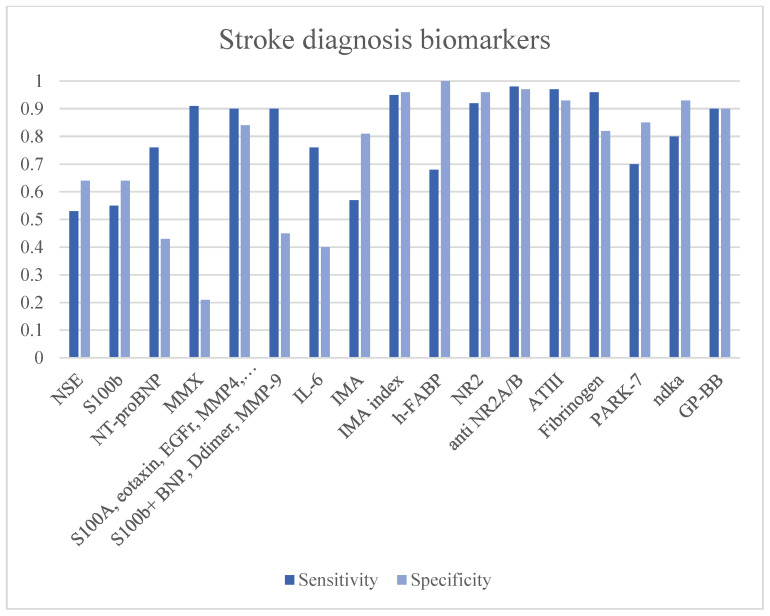
Stroke diagnosis biomarkers.

The S100b alone does not seem to have great diagnostic power and is mostly used in the differential between ICH and IS [39]. Good sensitivities with specificity < 50% have been achieved when associated with BNP, D-dimer and MMP9 [44,45]. S100A12 associated with Eotaxin, EGFR, MMP4 and prolactin showed good accuracy in the diagnosis of stroke [48]. NT-proBNP is a good biomarker in the diagnosis of stroke on a cardioembolic basis, thus allowing for an etiological assessment [55]. Figure 2 and Table 2.

### 3.12. Ischemic vs. Haemorrhagic Stroke Differential Diagnosis

Intracranial haemorrhage represents the main contraindication to revascularization therapy in stroke [5,6]. Making a prompt diagnosis even before the execution of imaging techniques could determine a shortening of the timing of intervention and, therefore, a reduction in post-stroke mortality and morbidity [1,3]. An optimal biomarker that helps rule out haemorrhagic lesions should be characterized by a high specificity to be sure that if a patient results negative to the test haemorrhagic lesion is not present in the first hours (<4.5 h).

**Figure 3:**
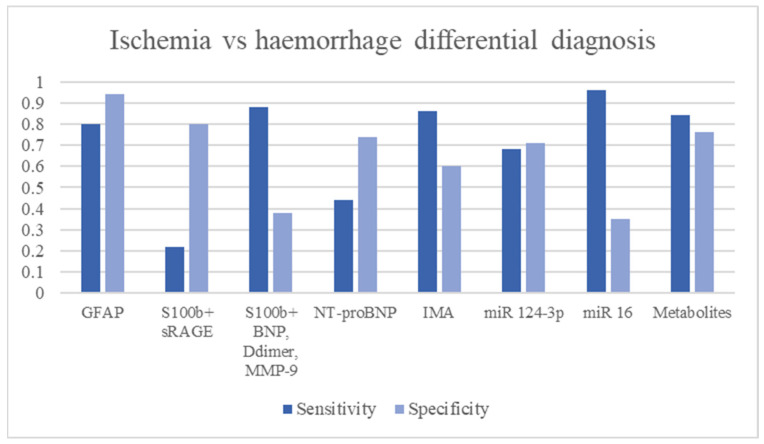
Ischemia vs. haemorrhage differential diagnosis biomarkers.

Among the biomarkers that distinguish these two types of strokes, GFAP appears to be the most studied. The rupture of the blood-brain barrier determines the apoptosis of astrocytes and the release of GFAP into the bloodstream [28]. Whereas in ischemia, such rupture occurs by depletion of ATP stores and dysfunction of the Na/K pump leading to apoptosis of the endothelium and astrocytes, in intracranial haemorrhage, there is a rapid and mechanical breakdown of the barrier [98]. This leads to a more rapid increase with higher concentrations of GFAP in intracranial haemorrhage than in ischemia [28]. Studies have shown high specificity and good sensitivity in the ICH/IS differential (specificity = 96.3–100%, LR+ 4.10–39.50, diagnostic OR: 23.11–326.93) [29,30,31,32]. The diagnostic accuracy seems to be directly proportional to the size of the hematoma, where for larger volumes of haemorrhage, there are greater increases in GFAP [30]. Association between GFAP and RBP4 showed worst diagnostic performance than GFAP alone (sensitivity 84%, LR+ 4.28, OR 11.36). The study of metabolomics and levels of amino acid metabolites in the blood through dried blood spot tests showed a difference in concentration in subjects with ischemic and haemorrhagic stroke [83]. The study results show good diagnostic accuracy of these biomarkers in the differential ICH/IS [83]. Good diagnostic performances were also achieved by S100b associated with RAGE [43] and by mi-RNA study [81]. In particular, mi-RNA124-3p showed good specificity in the differential between ischemic and haemorrhagic stroke [83]. (Figure 3 and Table 3).

### 3.13. Haemorrhagic Stroke Transformation Predictors

Haemorrhagic transformation of an ischemic stroke is a worse clinical outcome index leading to increased mortality and long-term disability [99]. Clinical factors associated with an increased risk of haemorrhagic transformation are stroke severity, advanced age, hypertension, hyperglycemia, early hypodensities on cerebral imaging, use of anti-thrombotic drugs and reperfusion therapies [99].

An optimal biomarker for predicting haemorrhagic transformation should be characterized by a high diagnostic odds ratio.

**Figure 4:**
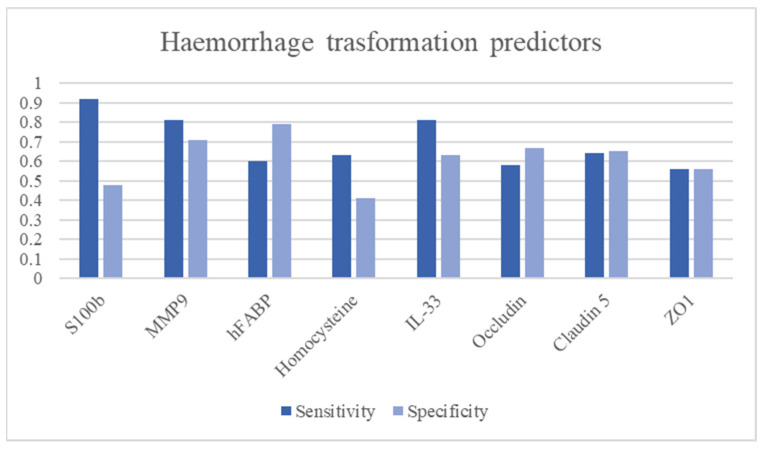
Haemorrhage stroke transformation predictors.

MMP-9 represents one of the actors of haemorrhagic transformation together with MMP-2 and ROS by participating in BBB rupture and subsequent blood extravasation [100]. Studies have shown good predictive power of haemorrhagic transformation (sensitivity: 68.7–85%, specificity: 45.3–81.3%, DOR: 1.82–21.08) [46,51,52]. Tigh junctions represent the mechanism that ensures the integrity of the BBB [101]. However, detecting an increase in tight junction components in the blood is not associated with a high predictive capacity for haemorrhage [46]. S100b showed good diagnostic performance as a negative predictive value of haemorrhagic transformation (sensitivity: 92.9%, specificity:48.1%, DOR 12.13) [46]. Reducing IL-33 levels in the blood may be a good predictive marker of haemorrhagic transformation(sensitivity: 81.3%, specificity:63%, DOR 7.40) [85]. Even NSE, which has not shown high diagnostic performance in discerning from stroke and mimics, may have a role in predicting haemorrhagic transformation. Studies have shown that a second peak in blood after that due to ischemia can predict impending BBB rupture and blood extravasation (DOR: 6.8) [38] Figure 4 and Table 4.

## 4. Discussion

Finding a biomarker or a biomarkers panel able to perform an early stroke diagnosis, differentiate an ischemic lesion from a haemorrhagic one and give prognostic information represents an extremely complex challenge. As mentioned above, the heterogeneity of stroke pathophysiology greatly influences the diagnostic accuracy of biomarkers. In this review, we analysed the needed parameters that an ideal biomarker should have according to the different purposes: high sensitivity, high likelihood ratio positive, high diagnostic odds ratio and early increase in blood.

The clinical utility of an early biomarker capable of diagnosing acute stroke and distinguishing between ischaemic and haemorrhagic stroke would significantly reduce the administration time of reperfusion therapies. Indeed, it would be possible to administer thrombolysis already in the outpatient clinic, increasing the effectiveness of therapy and improving short- and long-term outcomes. Conversely, a prognostic marker would allow the risk of haemorrhage to be determined and the timing of the initiation of chronic antiplatelet and anticoagulant therapies to be better identified.

During our analysis, however, no single biomarker was found to reflect all the characteristics sought.

For example, ATIII, fibrinogen and IMA index are biomarkers with very high diagnostic accuracy, even in the earliest stages of stroke. On the other hand, the studies conducted so far have shown no ability in stroke and haemorrhage differentiation nor the ability to predict haemorrhagic transformation. The opposite is true for GFAP, which has demonstrated good sensitivity and specificity in differentiating stroke and haemorrhage, especially when associated with large hematomas, but it cannot accurately determine between ischemic stroke and stroke mimics. MMP-9 showed a good predictive capacity for haemorrhagic transformation but low sensitivity in the differentiation between ischemic and haemorrhagic stroke and in stroke diagnosis. Based on this information, it does not seem possible to use a single biomarker for stroke, but it is necessary to create a panel of biomarkers with complementary characteristics in the diagnosis, differentiation, and prediction of stroke and outcomes.

### Biomarkers Panel

We proposed a panel based on four biomarkers with characteristics that allow comprehensive and early stroke diagnosis. The proposed biomarkers are ATIII, IMA index and fibrinogen for the diagnostic phase, GFAP, study of metabolomic and mi-RNA 124-3p for differentiation between ischemia and haemorrhage and MMP-9, S100b and IL-33 for prediction of haemorrhagic transformation.

These biomarkers reflect different stages and pathophysiological mechanisms underlying stroke. The decrease in ATIII levels and the increase in fibrinogen levels in the blood indicate endothelial damage following vessel thrombosis in ischemic stroke or vessel rupture in hemorrhagic stroke. Tissue ischemia triggers the release of reactive oxygen species, which are believed to be responsible for structural changes in albumin and the subsequent elevation of ischemia-modified albumin (IMA) levels. Although this increase occurs early, it may also be influenced by myocardial infarction, so it is crucial to assess ATIII levels, which are not associated with a reduction during myocardial infarction. GFAP serves as a biomarker for blood-brain barrier integrity. In cases of hemorrhagic stroke, the disruption of the blood-brain barrier leads to a more significant and rapid increase in GFAP levels in the blood. The impairment of nerve cells and the blood-brain barrier results in the release of tissue-specific miRNA 124-3p, which leads to an early increase in levels primarily observed in cerebral hemorrhage. MMP-9 indicates the activation of subendothelial matrix degradation processes, providing information about the state of the blood-brain barrier and the likelihood of rupture.

**Figure 5:**
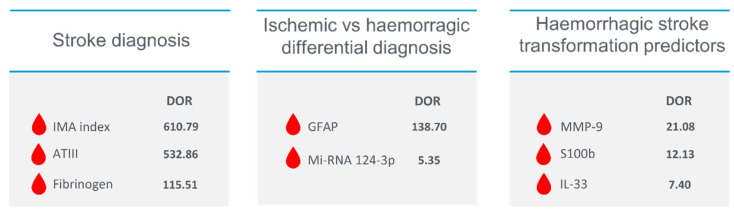
Stroke blood biomarkers panel.

Furthermore, the disruption of the BBB results in the release of the S100b protein from perivascular astrocytes. IL-33 is also present in endothelial cells, and elevated levels of IL-33 contribute to an increased risk of hemorrhagic transformation Figure 5.

## Figures and Tables

**Table 1:** Search strategy.

Domain	Search String
Cerebrovascular disease	(cerebrovascular disorder or brain vascular disorders or vascular diseases, intracranial or intracranial vascular disease or cerebrovascular occlusion or cerebrovascular accident or intracranial embolism, and thrombosis or cerebrovascular insufficiencies)
Ischemia	AND (ischemia or Stroke or infarction or brain infarction or hypoxia-ischemia or brain ischemia or ischemic attack)
Haemorrhage	AND (intracerebral haemorrhage, cerebral haemorrhage, or intracranial haemorrhage)
Biomarkers	AND (- a biological marker or biomarker or biologic marker or marker, biological, or biomarker panel)
Diagnosis	AND (blood plasma sample, serum plasma sample, cerebrospinal fluid, blood proteins, plasma, blood, marker, serum, or serum marker or laboratory markers) AND (diagnoses or diagnostic or examinations)
Accuracy	AND (accuracy or sensitivity or specificity or AUC)

**Table 2:** Summary of the features of stroke diagnosis biomarkers.

Name	Function	Aim	Time	Sample Size	Cut Off	Sensitivity	Specificity	LR+	LR−	Diagnostic OR	AUC	Ref.	Limitations
**NSE**	Glycolysis metalloenzyme. Marker of small cell carcinomas.	Stroke detection	8–48 h	79 patients (44 IS, 17 ICH, 11 mimics)	<14 mcg/L	53%	64%	1.47	0.73	2.00	0.73	[39]	
**S100b**	Glial protein with neurite extension, astrocyte proliferation and inhibition of microtubule assembly functions.	Stroke vs. TIA + Mimics	8–48 h	79 patients (44 IS, 17 ICH, 11 mimics)	<130 ng/L	55%	64%	1.53	0.70	2.17	0.60	[39]	
**S100b + BNP + D-dimer + MMP-9**		Stroke (all) diagnosis	<3 h	343 stroke	N/A	90%	45%	1.64	0.22	7.36	0.75	[44]	Low specificity in stroke diagnosis and differential.
		IS diagnosis				91%	45%	1.65	0.20	8.27	0.73		
**MMX (S100b + BNP + D-dimer + MMP-9)**		Stroke diagnosis	<6 h	196 (57 control, 89 IS, 11 ICH, 39 other brain disorders)	1.3	91%	21.5%	1.16	0.42	2.77	0.712	[45]	Low specificity in stroke diagnosis.
					5.9	20.2%	93.5%	3.11	0.85	3.64			
**S100A12 + eotaxin + EGFR + MMP4 + prolactin**		Stroke diagnosis	<4.5 h	167 (57 IS, 32 ICH, 41 TIA, and 37 mimics)		90%	84%	5.63	0.12	47.25		[48]	
**NT-proBNP**	a peptide secreted by cardiac ventricles in response to cardiomyocyte elongation with subsequent functions: increasing renal filtration rate and vasodilation.	Stroke type diagnosis (cardioembolic stroke)	<72 h	92 (66 IS 26 control)	265.5 pg/mL	71.4%	73.7%	2.71	0.39	7.00	0.77	[55]	Low accuracy in stroke diagnosis and differential. Good performance in stroke etiology diagnosis.
		IS with AF				94.4%	72.9%	3.48	0.08	45.35	0.92		
		Stroke diagnosis	<6 h	1308 patients (71.9% ischemic, 14.8% stroke mimics, and 13.3% haemorrhagic)	>3.47 ng/mL	76.9%	43.5%	1.36	0.53	2.56		[56]	
**IL-6**	Interleukin of inflammation	Stroke diagnosis	<6 h	1308 patients (71.9% ischemic, 14.8% stroke mimics, and 13.3% haemorrhagic)		76.8%	40.7%	1.30	0.57	2.27		[56]	
**IMA**	Albumin N-terminal portion modified by hypoxia, acidosis and ROS, resulting in a reduction of its metal-binding capacity	Stroke diagnosis	<3 h	118 (84 IS, 18 ICH, 16 TIA or epilepsy)	80 U/mL	57.8%	81.3%	3.09	0.52	5.95		[61]	Low performance in stroke diagnosis and differential
**IMA index**		Stroke diagnosis	< 3 h	52 (28 IS, 24 mimics)	98 U/mL	95.8%	96.4%	26.61	0.04	610.79	0.990	[63]	Not studied ischemic vs. haemorrhage differential performance.
**h-FABP**	Cytoplasmic proteins are involved in the metabolism of fatty acids, ensuring their transport to the mitochondrion for oxidation	Stroke diagnosis	<24 h	64 (22 strokes, 22 controls, 20 myocardial infarctions)	>0.531 (OD)	68.2%	100%	-	0.32	-		[65]	Low sensitivity in stroke diagnosis and differential.
**NR2**	Degradation product of NMDA receptor	Stroke diagnosis	<72 h	192 patients	1 mcg/L	92%	96%	23.00	0.08	276.00		[69]	Increase late after-stroke damage
**Anti-NR2A/B**	Antibodies against NMDA subunits	Stroke diagnosis	3–5 h ICH 9–12 h IS	460 (205 IS and ICH, 255 controls)	2.0 ng/mL	98%	97%	32.67	0.02	1584.33	0.99	[70]	Increase late after-stroke damage
		TIA diagnosis				98%	95%	19.60	0.02	931.00	0.99		
**ATIII**		Stroke diagnosis	<4.5 h	198 (152 IS, 46 mimics)	<210%	97.32%	93.62%	15.25	0.03	532.86		[72]	Not studied ICH vs. IS differential performances
**Fibrinogen**					>4 g/L	96.05%	82.61%	5.52	0.05	115.51			
**TNF alpha**	Systemic inflammation cytokin	Stroke prediction after carotid stenting	After surgery	255 (128 underwent endoartectomy)	9.45 pg/ml	45.3%	82.8%	2.63	0.66	3.99	0.651	[87]	Low performances in stroke prediction.
**PARK-7**	Oxidative stress response regulation protein	Stroke diagnosis	3 h	787 (622 IS, 165 controls)	9.33 mcg/L	54–91%	80–97%	6.30	0.31	20.29	0.74	[91]	Not studied stroke differential performance
**NDKA**	enzyme responsible for the catalysis of phosphate group exchange between different nucluoside diphosphate groups	Stroke diagnosis	3 h	787 (622 IS, 165 controls)	2 mcg/L	70–90%	90–97%	12.31	0.21	57.54	0.83		
**GP-BB**	the enzyme that catalyzes the degradation reaction of glycogen to glucose-1-phosphate	Stroke diagnosis	>4.5 h	305 (172 IS, 133 controls)	7.0 ng/mL	93%	93%	13.29	0.08	176.51	0.96	[97]	Need troponin I evaluation to improve specificity.

Legend: LR+: likelihood ratio positive, LR−: likelihood ratio negative, OR: odds ratio, ROS (reactive oxygen species), IS: ischaemic stroke, AF: atrial fibrillation, TIA: transient ischaemic attack, ICH: intracerebral haemorrhage.

**Table 3:** Summary of the features of ischemic vs. haemorrhagic stroke differential diagnosis.

Name	Function	Aim	Time	Sample Size	Cut Off	Sensitivity	Specificity	LR+	LR−	Diagnostic OR	AUC	Ref.	Limitations
**GFAP**	Astrocytes proteolytic enzime involved in cell-cell communication, astrocyte-neuron interaction, maintenaco of BBB, reparative process in CNS	Ischemia-Haemorrhage differential Hematoma size evaluation	<6 h	135 patients (42 ICH, 93 IS)	2.9 ng/L	79%	98%	39.50	0.21	184.33		[28]	Not able to diagnose IS
			<2 h	205 patients (39 ICH, 163 IS, 3 mimics)	2.9 ng/L	84.2%	96.3%	22.76	0.16	138.70		[29]	
			63 min (median)	74 patients (25 ICH, 49 IS)	2.9 ng/L	36% (hematoma volume < 15 mL)	100%	-	0.64	-		[30]	
						61.5% (hematoma volume > 15 mL)	100%	-	0.39	-			
			2–6 h	43 ICH 65 IS	0.7 ng/mL	86%	79%	4.10	0.18	23.11		[32]	
			<6 h	270 patients (IS: 121, ICH: 34, stroke mimics: 31, subarachnoid haemorrhage: 5, controls: 79)	0.43 ng/mL	91%	97%	30.33	0.09	326.93	0.97	[33]	
				Metanalyses		75–78%	95%	15.30	0.25	61.85	0.90–0.93	[34,35]	
**RBP4 + GFAP**		Ischemia-Haemorrhage differential		74 patients (25 ICH, 49 IS)	RBP4 > 48.75 mcg/mL GFAP > 0.7 ng/mL	68.4%	84%	4.28	0.38	11.36		[32]	
**S100b + sRAGE**	S100b receptor	Haemorrhage vs. ischemia	<3 h	915 (776 IS, 139 ICH)	S100b > 96 pg/mL sRAGE <0.97 ng/mL	22.7%	80.2%	1.15	0.96	1.19	0.762 (with the clinical feature)	[43]	Low sensitivity in ischemia-haemorrhage differential. Improved if it is associated with the patient’s clinical features
**S100b + BNP + D-dimer + MMP-9**		Haemorrhage diagnosis				88%	38%	1.42	0.32	4.49	0.81	[44]	
**NT-proBNP**	a peptide secreted by cardiac ventricles in response to cardiomyocyte elongation with subsequent functions: increasing renal filtration rate and vasodilation.	Haemorrhage vs. Ischemia	<6 h		>5.70 ng/mL	44.8%	74.9%	1.78	0.74	2.42		[56]	
**IMA**	Albumin N-terminal portion modified by hypoxia, acidosis and ROS, resulting in a reduction of its metal-binding capacity	ICH, IS, SAH diagnosis				86.8%	60.5%	2.20	0.22	10.07		[61]	
**Adrenomedullin**	Vasodilator peptide hormone	ICH diagnosis	<24 h	114 (50 controls, 64 ICH)	>69 pg/mL	80%	100%	-	0.20	-	0.89	[76]	
**miR 124-3p**	Non-coding RNA is involved in the regulation of gene expression, cell development, apoptosis and metabolism	ICH vs. IS	<24 h	93 (74 IS, 19 ICH)	>3 × 10^5^ copies/mL	68.4%	71.2%	2.38	0.44	5.35		[83]	Not studied in early ICH vs. IS differential. Not studied performance stroke diagnosis.
**miR 16**					≤2 × 10^9^ copies/mL	96.7%	35.1%	1.49	0.09	15.85			
**Metabolomics**	Small metabolites of lactate, pyruvate, glycolate etc.	ICH vs. IS	2–12 h	322 (129 IS, 128 ICH, 65 controls)		84%	76.9%	3.64	0.21	17.48		[83]	Not studied performance in stroke diagnosis.

Legend: LR+: likelihood ratio positive, LR−: likelihood ratio negative, OR: odds ratio, ROS (reactive oxygen species), IS: ischaemic stroke, AF: atrial fibrillation, TIA: transient ischaemic attack, ICH: intracerebral haemorrhage, SAH: subarachnoid haemorrhage.

**Table 4:** Summary of the features of haemorrhagic stroke transformation predictors.

Name	Function	Aim	Time	Sample Size	Cut Off	Sensitivity	Specificity	LR+	LR−	Diagnostic OR	AUC	Ref.	Limitations
**NSE**	Glycolysis metalloenzyme. Marker of small cell carcinomas.	Haemorrhagic transformation predictor Stroke volume		83 IS	Second peak			-	-	6.884		[38]	Low accuracy in stroke detection
**S100b**	Glial protein with neurite extension, astrocyte proliferation and inhibition of microtubule assembly functions.	Haemorrhagic transformation predictor	3–6 h	458 IS	>11.89 pg/mL	92.9%	48.1%	1.79	0.15	12.13	0.746	[46]	Low accuracy in stroke detection
**MMP-9**	A zinc-metalloproteinase is involved in extracellular matrix degradation.	Haemorrhagic transformation predictor	<24 h	168 IS	>181.7 ng/mL	82.9%	81.3%	4.43	0.21	21.08		[51]	Not able to diagnose stroke
			3–6 h	458 IS		68.7%	45.3%	1.26	0.69	1.82		[46]	
				1492 IS (metanalysis)		85%	79%	4.05	0.19	21.32	0.89	[52]	
**h-FABP**	Cytoplasmic proteins are involved in the metabolism of fatty acids, ensuring their transport to the mitochondrion for oxidation	Clinical outcome predictor	<24 h	238 (111 IS, 127 controls)	9.70 ng/mL	59.5%	79.5%	2.90	0.51	5.70	0.71	[66]	
**Homocysteine**	Methionine-derived amino acid	Haemorrhagic transformation		1378 patients	>16.56 μmol/L	63.3%	41.3%	1.08	0.89	1.21		[77]	Low specificity in haemorrhagic transformation
**IL-33**	Interleukin producted by T helper 2	Haemorrhagic transformation		191 (151 IS, 40 controls)	<67.66 ng/L	81.3%	63%	2.20	0.30	7.40	0.739	[85]	Not studied performance stroke diagnosis and differential
**Occludin**	Blood-brain barrier tight junction components	Haemorrhagic transformation	<3 h	458 IS	>0.029/ng/mL	58.6%	67.5%	1.80	0.61	2.94	0.622	[46]	Not able to diagnose stroke
**Claudin 5**					1.601 ng/mL	64.3%	65.8%	1.88	0.54	3.47	0.599		
**ZO1**					>1.36 RU/mL	56.7%	56%	1.29	0.77	1.67	0.519		

Legend: LR+: likelihood ratio positive, LR−: likelihood ratio negative, OR: odds ratio, ROS (reactive oxygen species), IS: ischaemic stroke, AF: atrial fibrillation, TIA: transient ischaemic attack, ICH: intracerebral haemorrhage, SAH: subarachnoid haemorrhage.

## Data Availability

Not applicable.
